# Effects of trimodal prehabilitation on quality of life and negative emotions in patients with advanced triple-negative breast cancer

**DOI:** 10.3389/fonc.2026.1750574

**Published:** 2026-05-01

**Authors:** Rudan Deng, Enhui Liu, Juan Zhou, Qingqing Zhao, Linyun Pan, Yong Liu, Yang Yu

**Affiliations:** 1 The Affiliated Xuzhou Clinical College of Xuzhou Medical University, XuZhou, China; 2 Department of Medical Oncology, Xuzhou Central Hospital, XuZhou, China; 3 Department of Laboratory Medicine, Xuzhou Central Hospital, XuZhou, China; 4 Xuzhou Medical University, XuZhou, China

**Keywords:** cancer-related fatigue, negative emotions, progression-free survival, quality of life, trimodal prehabilitation, triple-negative breast cancer

## Abstract

**Objective:**

To assess the effectiveness of a four-week trimodal prehabilitation program in enhancing the quality of life, clinical symptoms, and negative emotions of stage IV triple-negative breast cancer patients who are undergoing first-line systemic therapy. Optimize comprehensive care management for advanced cancer patients by providing evidence-based guidance.

**Methods:**

Ninety-seven patients with stage IV triple-negative breast cancer at Xuzhou Central Hospital were selected from January 2024 to June 2025, and categorized into an observation group (49 cases) and a control group (48 cases). The control group underwent standard anti-tumor treatment and routine care, whereas the observation group engaged in a 4-week trimodal prehabilitation intervention in conjunction with standard treatment. The quality of life of patients, along with cancer-related fatigue, pain, anxiety, and depression, was evaluated using the Functional Assessment of Cancer Therapy-Breast (FACT-B), the Revised Piper Fatigue Scale (PFS-R), the Numerical Rating Scale (NRS), the Hamilton Anxiety Rating Scale (HAMA), and the Hamilton Depression Rating Scale (HAMD) prior to and following the intervention. Patients in two groups were followed up after the completion of treatment to record their progression-free survival (PFS).

**Results:**

Following the intervention, the observation group had significantly higher scores on the FACT-B scale (112.16 ± 4.67) than the control group (P < 0.001). They experienced considerably less cancer-related fatigue (5.33 ± 0.72) than the control group (P < 0.001), but there was no significant difference in pain scores between the two groups (P > 0.05). The observation group demonstrated considerably reduced levels of anxiety (13.14 ± 4.14) and depression (10.65 ± 3.48) compared to the control group (P < 0.001). In this trial, we preliminarily observed a longer PFS in the observation group compared with the control group (298.00 vs 235.00, P = 0.0237), along with a favorable safety profile.

**Conclusion:**

Among patients with stage IV triple-negative breast cancer receiving first-line systemic therapy, a 4-week trimodal prehabilitation intervention was associated with significantly improved quality of life, reduced cancer-related fatigue, anxiety, and depression. It is possible that the improvement in quality of life may translate into a survival benefit.

## 1 Introduction

Breast cancer is the second most prevalent malignant tumor worldwide as of 2022, and has become the most prevalent cancer among women in terms of incidence and mortality. The incidence rate of cancer is increasing, and the burden of the disease is gradually shifting from men to women, according to data (1). The survival period for breast cancer patients has been substantially extended as a result of the widespread adoption of early screening and the advancements in diagnostic and treatment techniques (2). Nevertheless, the quality of life and clinical outcomes of advanced patients are significantly impacted by physical decline, malnutrition, and psychosocial adaptation disorders, which are a result of disease progression and the effects of multiple lines of anticancer treatment (3, 4). In this context, trimodal prehabilitation, which integrates psychological support, nutritional intervention, and exercise training, has progressively emerged as a prominent area of research. This paradigm is an extension of the Enhanced Recovery After Surgery (ERAS) concept, which emphasizes the development of the body’s functional reserves to improve the patient’s capacity to adapt to “stressful events” such as illness or treatment (5). Initially, the clinical application of exercise prehabilitation was predominantly focused on patients who were undergoing cardiac surgery and joint replacement surgery (6). However, as research has advanced, numerous clinical studies have shown that multi-modal prehabilitation programs produce more substantial clinical outcomes than single-modal intervention strategies (7). The triad model of exercise, psychology, and nutrition has progressively become a primary focus in clinical practice and research as a result of this discovery. In 2013, Julie K. Silver introduced the concept of “cancer rehabilitation” which specifically refers to the continuous care process from the diagnosis of cancer to the initiation of acute treatment. This process entails the establishment of baseline functional levels, the identification of impairments, and the provision of targeted interventions that are designed to enhance the overall health of patients and decrease the occurrence and severity of current and future impairments (8). At present, evidence-based research has tentatively validated the value of trimodal prehabilitation in the perioperative management of solid tumors, including colorectal cancer and gynecological tumors (9, 10). However, it remains in the exploratory phase in the field of breast cancer. Current studies predominantly concentrate on preoperative rehabilitation for early-stage breast cancer, intending to minimize the postoperative rehabilitation duration and mitigate consequences (11). Nevertheless, the primary physical burdens for patients with advanced breast cancer are pain, fatigue, and treatment side effects, which substantially impair their health-related quality of life and physical functioning (12). As a result, intervention strategies for these patients should prioritize the improvement of treatment tolerance, the postponement of functional decline, and the enhancement of their overall quality of life. Trimodal prehabilitation is slowly gaining recognition in the field of cancer rehabilitation, however, its core mechanisms of action, specific implementation protocols, and feasibility remain uncertain for patients with advanced-stage cancers, particularly those with a poor prognosis and high treatment toxicity, such as triple-negative breast cancer. Therefore, it is critically important to comprehensively examine the intervention effects and implementation strategies of the trimodal prehabilitation program for patients with advanced breast cancer to enhance overall cancer care.

## 2 Objects and methods

### 2.1 Objects

#### 2.1.1 Selection of research subjects

This was a prospective, two-arm randomized controlled trial that ran from January 2024 to June 2025. This study’s subjects were patients with stage IV Triple-negative breast cancer from a tertiary hospital. The participants were randomly assigned to one of two groups: observation or control.

#### 2.1.2 Inclusion and exclusion criteria

Inclusion criteria: (1) female, aged 18–75 years old; (2) pathologically confirmed diagnosis of Triple-negative breast cancer (including initial diagnosis and primary treatment, or early surgery and metastasis in postoperative follow-up); (3) planned to receive standard systematic antitumor therapy; (4) no obvious contraindications to treatment in electrocardiogram, liver and kidney function, and blood routine tests; (5) no diseases such as joint mobility disorder.

Exclusion criteria include: (1) patients with severe cardiac, hepatic, renal diseases, or other malignant tumors; (2) patients with a history of mental illness, cognitive dysfunction, or intellectual disability who are unable to express their conditions accurately; (3) patients who have had cardiovascular or cerebral vascular accidents within the last 6 months; (4) patients with poor nutritional status who require nutritional support via nasogastric tube or parenteral nutrition; (5) patients whom the investigator deems unsuitable for participation in the study.

The study excluded patients who withdrew on their own, had a prehabilitation completion rate of less than 60%, or had missing data due to treatment abandonment, transfer to another facility, or failure to complete the 1/4 intervention course.

#### 2.1.3 Sample size calculation

In this study, the FACT-B score was used as the primary outcome for sample size estimation. Based on previous research, the anticipated difference in the mean total FACT-B score between the two groups after treatment was 8 points (13). The significance level was set at α = 0.05 (two-tailed test), and the statistical power was set at 1 – β = 0.90. Sample size was calculated using G*Power software, which yielded approximately 39 patients needed per group. Allowing for a 20% dropout rate, we planned to enroll 50 patients per group, for a total sample size of 100.

#### 2.1.4 Grouping method

Using SPSS 27.0, we generated a random sequence of 100 random numbers and recorded the serial number for each. Participants were then assigned to groups based on the size of the random numbers: those ranked 1 to 50 were placed in the observation group, and those ranked 51 to 100 were placed in the control group. The allocation table, which contained the serial numbers, random numbers, and group labels, was sealed and stored in an opaque envelope. When enrolling participants according to the inclusion criteria, they were assigned a sequential number based on their order of visit and allocated accordingly. In the end, there were 50 participants in both the control and intervention groups.

### 2.2 Intervention method

#### 2.2.1 Intervention methods in the control group

Patients underwent routine medical examinations, daily health education, basic nutrition advice, exercise supervision, and routine psychological therapy. No structured exercise regimen, systematic psychological intervention, or personalized nutritional support was implemented. Standardized antitumor therapy was implemented in accordance with the guidelines: Taxane monotherapy, taxane-based combination chemotherapy, or chemotherapy combined with PD-1 inhibitor immunotherapy, concurrent denosumab therapy for those with bone metastases, and close monitoring and timely management of treatment-related adverse reactions. The assessment time nodes for patients’ quality of life, clinical symptoms, and negative emotions matched those of the observation group.

#### 2.2.2 Intervention methods of the observation group

In addition to standardized anti-tumor treatment in the control group, a 4-week trimodal prehabilitation intervention was implemented. During the first week, the intervention was delivered by the researcher under hospital supervision. Patients performed the intervention at home during weeks two to four, and the entire treatment was remotely overseen. To ensure that the intervention was completed, patients clocked in and provide feedback on their exercise duration, psychological training completion status, and dietary records daily via WeChat or phone. Graduate students verify the daily clock-in records, proactively urge patients who fail to clock in, record the reasons, and provide targeted guidance. They conduct follow-up visits twice a week, meticulously documenting patients’ intervention progress, physical reactions, and actual problems encountered, and promptly adjusting the intervention plan. The researcher synchronously recorded the above information in the follow-up logbook to monitor training progress in real time and oversee those who did not reach the criteria, confirming the intervention’s efficacy and data validity. The precise intervention material is as follows.

##### 2.2.2.1 Exercise prehabilitation

The researcher evaluated the patients’ physical capabilities and formulated a customized training program that integrates personal preferences. The program incorporated aerobic workouts, including brisk walking, water walking, and stationary cycling, performed 3–5 times weekly for 20–30 minutes each session. The intensity and duration are adjusted based on the degree of fatigue. Flexibility training comprises adapted yoga and seated stretching sessions conducted 3–5 times weekly for 15–20 minutes each, in addition to deep breathing exercises. Engage in resistance exercise 2–3 times weekly, utilizing elastic bands, light dumbbells, or bodyweight squats, performing four sets of 10–15 reps each. The workout intensity adheres to a progressive load increment pattern, targeting a “slight degree of exertion” as rated by the patient. Patients quantified their heart rate by wearing wearable devices or using the Rating of Perceived Exertion (RPE) scale, with the heart rate controlled at (220 - age) × (70-80%) beats per minute. Before the exercise intervention, all patients underwent assessments of bone metastasis sites, lesion types, stability, and fracture risk, and were stratified and managed according to low, medium, and high risks. Patients with high-risk bone metastasis (spine, femoral neck, proximal humerus) were absolutely prohibited from weight-bearing and standing walking exercises. Patients with medium-risk bone metastasis were prioritized to choose non-weight-bearing exercises such as water exercise and seated cycling, and were prohibited from jumping, twisting, and weight-bearing resistance exercises. Patients with low-risk bone metastasis could implement conventional exercise programs supplemented with light resistance training. If symptoms such as nausea, vomiting, fatigue, dizziness, and chest pain occurred, the exercise was immediately terminated and recorded. The exercise program was adjusted after the team assessed tolerance.

##### 2.2.2.2 Psychological prehabilitation

After admission, patients were assessed for psychological distress using the Distress Thermometer (DT). Interventions were then implemented based on the assessment results. First, the researcher provided targeted education to patients, which covered the psychological characteristics of advanced breast cancer, self-regulation techniques for negative emotions, and the importance of family support. This education helped patients develop a realistic understanding of the disease, accept their emotional responses, and reduce psychological distress. Next, the researcher guided patients in relaxation techniques such as mindfulness meditation and music therapy. Patients could choose either technique or a combination based on personal preference, practicing at least four times per week for 20 minutes each session. During the first week of hospitalization, the researcher led patients through daily relaxation sessions to ensure they mastered the essential skills. After discharge, patients practiced independently at home by watching professional instructional videos or listening to audio recordings. It should be noted that the researchers who guided the relaxation training had all completed specialized training in psychological therapy and were proficient in the relaxation techniques of mindfulness-based stress reduction and music therapy. The DT was administered weekly to reassess the level of psychological distress, and intervention strategies were adjusted according to the patient’s DT score. For patients with a score of 3 or lower, the existing intervention was maintained. For those scoring between 4 and 6, individualized psychological counseling was provided by a psychotherapist. For patients with a score of 7 or higher, a multidisciplinary team consultation was initiated immediately, and targeted intervention was developed in collaboration with the psychology department if needed.

##### 2.2.2.3 Nutritional prehabilitation

Patients were encouraged to maintain a balanced diet rich in high-quality proteins, carbohydrates, fruits, and vegetables. Daily protein intake should range from 1.2-1.5g per kilogram per day, accompanied by a caloric consumption of 35–40 kcal per kilogram per day. The Nutrition Risk Screening 2002 (NRS 2002) was utilized to evaluate patients’ nutritional risk, and the researchers implemented individualized adjustments based on the screening outcomes. An NRS score below 3 signifies that no further nutritional intake is required, but weekly nutritional evaluation is essential. For patients with an NRS score of 3 or higher, meeting nutritional needs through food is the first choice. In addition, enteral nutritional supplements may be used depending on the patient’s condition. These patients should consume an additional 400–600 kcal per day, with protein providing at least 20% of the total energy from this additional nutritional intake. The extra nutrition should be taken in two to three divided doses, either with meals or between meals. Furthermore, patients must receive dietary guidance to help change unhealthy eating habits and preferences, and smoking and alcohol intake should be strictly limited. For patients who opted to meet their nutritional needs through food, the researchers prepared a quantified food table in advance. Protein was preferably obtained from eggs or milk. For example, one egg contains approximately 80 kcal, with protein contributing about 25 kcal. One hundred milliliters of whole milk contains about 65 kcal, with protein contributing about 12 kcal. Reference calorie values for other common foods were as follows: one cooked corn (approximately 170 kcal), one banana (approximately 90 kcal), a fist-sized portion of fish (approximately 100 kcal), a fist-sized portion of rice (approximately 110 kcal), and a fist-sized portion of vegetables (approximately 25 kcal), and so on. We recommended that patients consume at least two eggs and one 250-mL bottle of whole milk daily. The remaining calories could be supplemented according to the reference values provided in the quantified table. Because variations in food preparation and brand exist, complete precision in quantification was not possible. Therefore, the researchers provided individualized guidance based on the patients’ daily food logs to ensure that their calorie intake met the trial requirements. Patients who chose Oral Nutritional Supplements (ONS) received an Intacted Protein Enteral Nutrition Powder (brand name: Nutrison; specification: 320 g/1478.4 kcal per can). They took one-third of a can, dissolved it in 500 mL of water, and consumed it in divided doses either with meals or between meals.

### 2.3 Observation indicators

#### 2.3.1 Quality of life

The Functional Assessment of Cancer Therapy-Breast (FACT-B) scale was used to assess patients’ quality of life. The FACT-B scale comprises five dimensions with 36 items: physical status (7 items), social/family status (7 items), emotional status (6 items), functional status (7 items), and additional breast cancer concerns (9 items). The assessment utilized a five-point Likert scale (0-4), yielding a total score range of 0-144; elevated scores signify an improved quality of life for the patient. The assessment of quality of life was conducted at week 1 and week 4.

#### 2.3.2 Clinical symptoms

The evaluated clinical symptoms comprise cancer-related fatigue and cancer-related pain. Cancer-related fatigue was evaluated utilizing The Revised Piper Fatigue Scale (PFS-R), which has a 0–10 point scale. A score of 0 signifies the absence of exhaustion, whereas a score of 10 denotes extreme fatigue; rising scores reflect increased severity of fatigue. Cancer-associated pain was evaluated with the Numerical Rating Scale (NRS), which has a scoring range of 0 to 10. Elevated scores signify increased pain severity.

#### 2.3.3 Negative emotions

Negative emotions, including anxiety and depression, were evaluated with the Hamilton Anxiety Rating Scale (HAMA) and the Hamilton Depression Rating Scale (HAMD), respectively. The HAMA comprises 14 items that address two dimensions: physical anxiety and mental anxiety. Each item is evaluated on a five-point scale from 0-4, yielding a cumulative score range of 0-56. The HAMD comprises 17 items that address three dimensions: emotional symptoms, physical symptoms, and cognitive and behavioral symptoms. Each item is evaluated on a five-point scale from 0-4, yielding a cumulative score range of 0-54. Elevated scores signify increased severity of anxiety and depression. The evaluation of unpleasant emotions is aligned with the assessment of quality of life. Assessment of patients’ negative emotions was performed by doctors who held a social work certificate and were qualified to provide psychological counseling.

### 2.4 Data analysis

Statistical analysis was conducted with the SPSS 27.0 program. Measurement results were presented as mean ± standard deviation (x ± s). For comparisons between two groups, an independent samples t-test was applied, while paired samples t-tests were used for within-group comparisons before and after the intervention. Categorical variables such as gender, age, and education level were analyzed using the chi-square (x^2^) test or rank-sum test. Survival graphs were plotted using Kaplan-Meier, and patient survival was tested by log-rank. Statistically significant differences were observed at P < 0.05.

## 3 Results

### 3.1 Subject enrollment status

**Figure 1:**
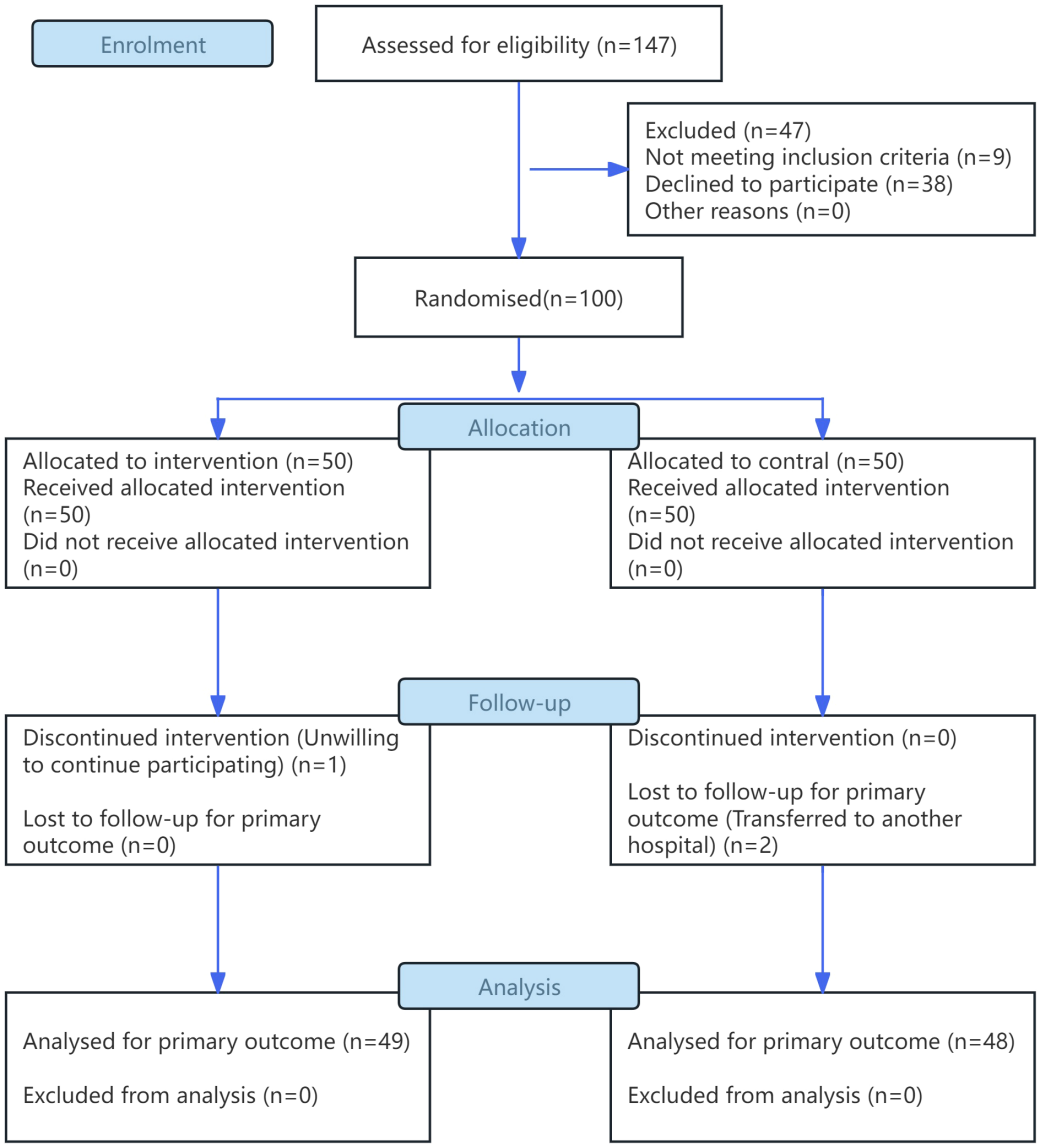
CONSORT flow diagram.

Based on the estimated sample size, 100 patients were recruited and randomly assigned using simple randomization to an observation group (n = 50) or a control group (n = 50). During the study, three patients withdrew, resulting in a dropout rate of 3%. Specifically, one patient from the observation group withdrew because of unwillingness to continue the intervention, and two patients from the control group withdrew and were lost to follow-up because they transferred to other hospitals for treatment. As a result, 97 patients completed the trial: 49 in the observation group and 48 in the control group. All 97 enrolled patients completed the full 4-week intervention, and the adherence rate for each of the three rehabilitation components was ≥60%. Therefore, the patients included in the intention-to-treat analysis were the same as those in the per-protocol analysis. (**Figure 1**).

### 3.2 Comparison of general information between the two patient groups

There were no statistically significant differences in baseline data, including educational level, marital status, bone metastasis status, treatment regimen, time of metastasis, BMI, and KPS score, between the two groups (P > 0.05). (**Table 1**).

**Table 1:** Comparison of baseline characteristics between the 2 groups.

Characteristics	Categories	Observation group (n=49)	Control group (n=48)	Statistical value	P
Age(years old)		57.94 ± 3.00	57.90 ± 2.90	t=0.072	0.943
Educational level	Primary and below	7(14.3%)	6(12.5%)	X^2^ = 0.377	0.945
	Junior high school	15(30.6%)	14(29.2%)		
	Senior high school	20(40.8%)	19(39.6%)		
	College and above	7(14.3%)	9(18.8%)		
Marriage	Married	43(87.8%)	41(85.4%)	X^2^ = 0.114	0.735
	Divorcee	6(12.2%)	7(14.6%)		
Bone metastasis	Yes	15(30.6%)	17(35.4%)	X^2^ = 0.253	0.615
	No	34(69.4%)	31(64.6%)		
Treatment plan	Chemotherapy	18(36.7%)	14(29.2%)	X^2^ = 0.628	0.428
	Chemotherapy combined with immunotherapy	31(63.3%)	34(70.8%)		
Transfer time	First visit	5(10.2%)	2(4.2%)		
	After treatment	44(89.8)	46(95.8%)	X^2^ = 1.320	0.251
BMI		23.15 ± 2.66	23.91 ± 3.09	t=1.301	0.196
KPS		81.63 ± 6.24	81.67 ± 6.30	t=−0.027	0.979

### 3.3 Comparison of the quality of life before and after treatment

Upon admission, no statistically significant difference in FACT-B scores was seen between the two groups (P > 0.05). Following four weeks of treatment, the observation group exhibited marked enhancements in physical, social, familial, emotional, functional, breast cancer-specific statuses, and overall scores in comparison to pre-treatment levels and the control group (P < 0.05). Conversely, the control group demonstrated no significant alterations in scores pre- and post-treatment (P > 0.05). (**Table 2**).

**Table 2:** Changes in FACT-B scores before and after treatment between 2 groups.

Groups	*N*	Time	Physical well-being	Social/family well-being	Emotional well-being	Functional well-being	Breast cancer subscale	Total score
Observation group	49	Before treatment	10.55 ± 2.63	13.16 ± 2.82	6.24 ± 2.06	11.10 ± 3.11	19.49 ± 2.41	60.55 ± 5.70
		After treatment	23.35 ± 2.30ab	17.90 ± 2.49ab	18.63 ± 1.99ab	20.24 ± 2.93ab	32.04 ± 2.59ab	112.16 ± 4.67ab
		t/P	−54.860/ <0.001	−14.774/ <0.001	−69.081/ <0.001	−46.228/ <0.001	−43.028/ <0.001	−87.752/ <0.001
Control group	48	Before treatment	8.83 ± 1.71	13.81 ± 2.83	7.63 ± 2.06	12.60 ± 2.89	17.54 ± 1.35	60.42 ± 5.43
		After treatment	9.25 ± 1.68	14.08 ± 2.53	8.25 ± 2.29	12.46 ± 2.67	17.88 ± 2.08	61.92 ± 4.16
		t/P	−1.360/0.180	−0.497/0.621	−1.524/0.134	0.295/0.769	−1.008/0.319	−1.703/0.095
Comparison between group(t/P)		After treatment	34.541/ <0.001	7.486/ <0.001	23.854/ <0.001	13.655/ <0.001	29.668/ <0.001	55.972/ <0.001

Compared with pre-treatment, aP < 0.05; compared with control group, bP < 0.05.

### 3.4 Comparison of clinical symptoms before and after treatment

The observation group exhibited a considerable reduction in cancer-related fatigue compared to pre-treatment levels and the control group, with statistically significant differences in scores (P < 0.05). In the control group, there were no significant changes in scores from before to after treatment (P > 0.05). The observation group exhibited a marginal decrease in cancer-related pain scores relative to pre-treatment levels; however, no statistically significant differences were seen when compared to pre-treatment levels or the control group (P > 0.05). (**Table 3**).

**Table 3:** Changes in PFS-R and NRS scores before and after treatment between 2 groups.

Groups	n	PFS-R				NRS			
		Before treatment	After treatment	t	P	Before treatment	After treatment	t	P
Observation group	49	8.40 ± 0.84	5.33 ± 0.72ab	26.276	<0.001	6.29 ± 1.74	5.84 ± 1.57	1.355	0.182
Control group	48	8.42 ± 0.73	8.36 ± 0.66	0.502	0.618	6.83 ± 1.86	6.31 ± 1.72	1.428	0.160
t		−0.133	−21.562			−1.496	−1.424		
P		0.895	<0.001			0.138	0.158		

Compared with pre-treatment, aP < 0.05; compared with control group, bP < 0.05.

### 3.5 Comparison of negative emotions before and after treatment

Following four weeks of treatment, the anxiety and depression scores in the observation group were markedly reduced compared to both their pre-treatment scores and those of the control group (P < 0.05). Conversely, no statistically significant difference was observed in the anxiety and depression scores among the control groups (P > 0.05). (**Table 4**).

**Table 4:** Changes in HAMA and HAMD scores before and after treatment between 2 groups.

Groups	n	HAMA				HAMD			
		Before treatment	After treatment	t	P	Before treatment	After treatment	t	P
Observation group	49	19.57 ± 4.63	13.14 ± 4.14ab	7.308	<0.001	18.43 ± 4.49	10.65 ± 3.48ab	20.932	<0.001
Control group	48	19.88 ± 4.68	19.58 ± 4.33	0.369	0.714	17.85 ± 4.08	17.46 ± 4.00	1.640	0.108
t		−0.321	−7.489			0.659	−8.946		
P		0.749	<0.001			0.511	<0.001		

Compared with pre-treatment, aP < 0.05; compared with control group, bP < 0.05.

### 3.6 Progression-free survival graphs of two groups

**Figure 2:**
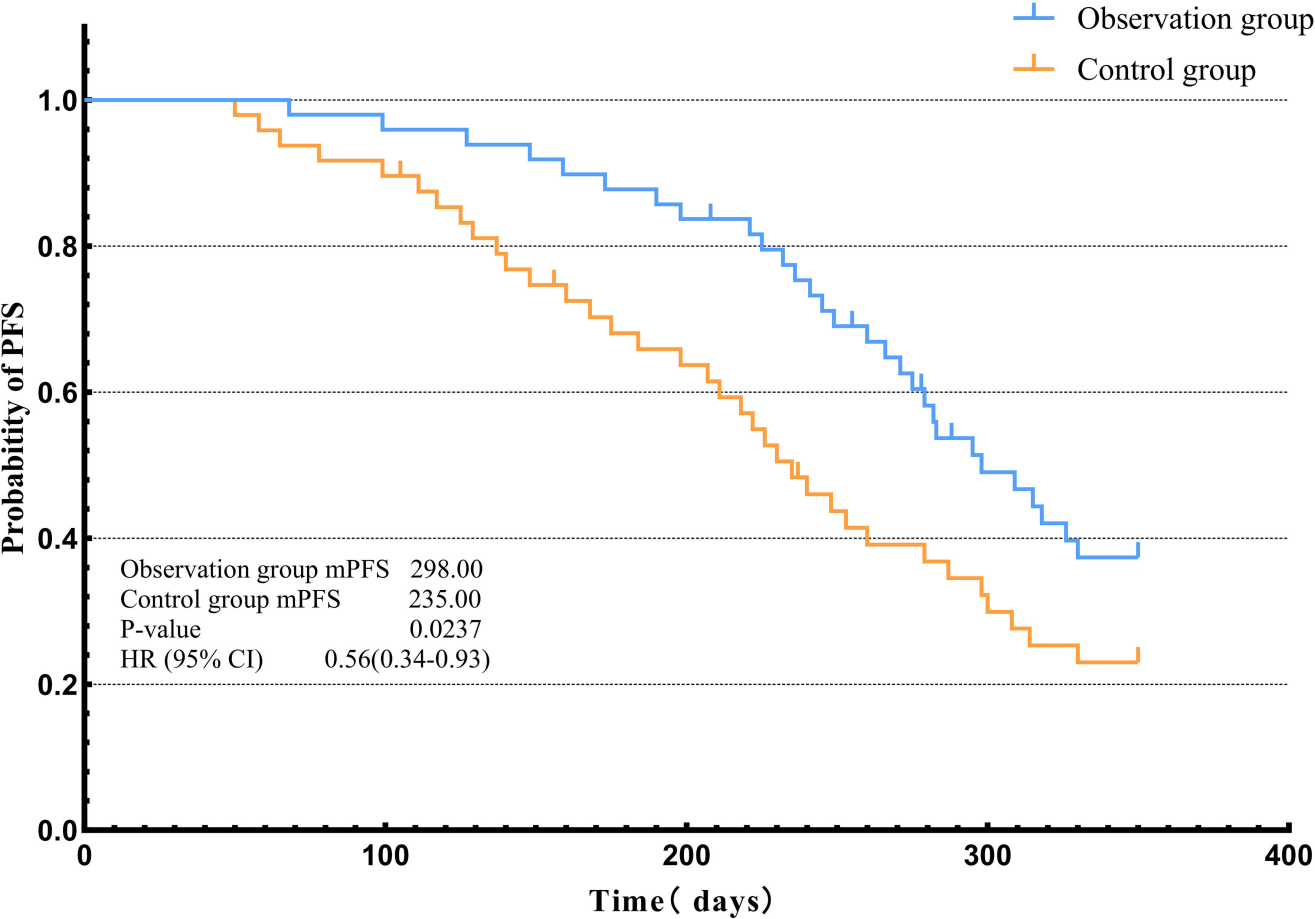
Kaplan‑Meier analysis of progression‑free survival (PFS) in the two patient groups.

No mortalities were recorded among the patients throughout the duration of the study. Kaplan-Meier survival analysis was performed on the advanced triple-negative breast cancer patients in the 2 groups, and it was found that the progression-free survival of the observation group was longer than that of the control group, with a median PFS of 235.00 days and 298.00 days (P = 0.0237). (**Figure 2**).

### 3.7 Incidence of adverse events

No serious adverse events occurred throughout the study. There were no statistically significant differences between the two groups in the incidence of nausea and vomiting, myelosuppression, liver function impairment, peripheral neurotoxicity, muscle soreness, or psychological stress reactions (P > 0.05). (**Table 5**).

**Table 5:** Incidence of adverse events in the two patient groups.

Classification of adverse events	Observation group [*n*(%)]	Control group [*n*(%)]	*χ* ^2^	*P*
nausea and vomiting	31(63.27)	36(75.00)	1.563	0.211
myelosuppression	22(44.90)	25(52.08)	0.501	0.479
liver function impairment	3(6.12)	2(4.17)	-	1.000
Peripheral neurotoxicity	2(4.08)	2(4.17)	-	1.000
muscle soreness	2(4.08)	0	-	0.495
Psychological stress response	1(2.04)	0	-	1.000

## 4 Discussion

As a result of the combined effects of disease progression and treatment interventions, patients with advanced breast cancer frequently experience multifaceted health crises. Metabolic disorders induced by tumor cells result in a high-energy consumption state in the body (14), whereas chemotherapy induces immune suppression and toxic side effects (15). Furthermore, patients’ physical reserves undergo a continuous decline as a result of protracted immobilization, which induces sarcopenia (16). On a subjective level, fear of cancer recurrence is significantly exacerbated by uncertainty regarding the disease prognosis (17), postoperative body image changes induce psychological distress, including anxiety and depression (18), and the body’s elevated inflammatory state further exacerbates CRF (19). The intricate nature of the injury in this bio-psycho-social medical model restricts the efficacy of conventional single-mode rehabilitation interventions. Physical exercise alone is unable to remedy nutritional substrate deficiencies, psychological counseling is unable to reverse abnormal catabolic processes, and nutritional support is unable to directly regulate psychological stress responses. In light of this context, this study pioneered the application of a perioperative “exercise-nutrition-psychological” trimodal prehabilitation paradigm to advanced triple-negative breast cancer patients who were undergoing first-line treatment. The applicability and efficacy of this model in the specific population of advanced triple-negative breast cancer patients remain unexplored, despite its validation in early-stage breast cancer. Our results verify that a structured, family-based triple-pronged approach is not only implementable but also considerably enhances the quality of life of patients by effectively alleviating core symptoms, including anxiety, depression, and fatigue related to cancer. This establishes a new paradigm for holistic rehabilitation in this patient population that is both robust and effective.

Despite the fact that the intervention models and implementation durations of existing prehabilitation studies for breast cancer patients are significantly heterogeneous, there is a widespread consensus regarding the clinical benefits of trimodal prehabilitation for breast cancer patients. By regulating patients’ physiological and psychological axes in multiple dimensions, various rehabilitation models have been able to substantially enhance quality of life and physical function, as well as key indicators such as negative emotions. This has been confirmed by numerous studies (20). Faul recorded the baseline levels of physical activity and quality of life of 192 patients with varying diagnoses and cancer stages. The findings indicated that a substantial improvement in overall quality of life and alleviation from anxiety and depression were observed in two-thirds of patients who engaged in physical activity prior to chemotherapy, as opposed to those who did not (21). We found that trimodal prehabilitation improves quality of life in patients with advanced triple-negative breast cancer, particularly in the domains of physical health and emotional well-being. The underlying reason may lie in the synergistic effects of exercise, psychological support, and nutrition, which go beyond a simple addition of individual components. Resistance training promotes muscle protein synthesis, increases lean body mass and mitochondrial density, and effectively counteracts cancer-related sarcopenia induced by chemotherapy (22). Aerobic exercise enhances oxygen-carrying capacity, improves cardiorespiratory fitness, and boosts physical performance, leading to significant improvements in patients’ physiological status (23). The combination of both exercise types is superior to either alone, exerting protective effects by regulating inflammatory factors, improving insulin sensitivity, and accelerating chemotherapy drug metabolism (24). Psychological support alleviates negative experiences by modulating stress responses and emotional pathways. Mindfulness-based stress reduction (MBSR) helps patients become aware of their own state and reduces anxiety and fear (25). Studies have shown that six to eight weeks of mindfulness intervention can increase telomerase activity (26), and long-term practice may alter brain structures, such as enhancing left-sided neural activity, reducing gray matter density in the amygdala, and increasing hippocampal volume, thereby maintaining internal homeostasis (27). Five-element music therapy, grounded in traditional Chinese medicine theory, coordinately regulates emotions and organ function, complementing mindfulness meditation (28). Nutritional therapy addresses the decreased appetite and hypercatabolic state characteristic of advanced breast cancer by supplementing high-quality protein and calories, maintaining nutritional parameters, and reducing weight loss and muscle wasting (29). Anti-inflammatory foods can also improve the inflammatory microenvironment and alleviate symptoms such as pain.

The incidence of CRF in breast cancer patients is higher than that in patients with other cancer types (30). To date, no unified conclusion has been reached regarding the pathogenesis of CRF; however, cytokine dysregulation is widely recognized as one of the fundamental mechanisms both in China and internationally (31). In addition, studies have shown that negative emotions, including anxiety and depression, are closely associated with CRF in breast cancer patients (32). The results of this study indicate that trimodal prehabilitation intervention significantly alleviates CRF in participants while also effectively relieving various negative emotions. Several factors may explain these findings. Mindfulness-based therapy guides patients to accept their difficulties, enhances psychological resilience, and reduces anxiety and depression (33). Five-element music therapy, through sound wave resonance, regulates qi flow, balances yin and yang, modulates autonomic nervous function and stress hormones, and helps break negative thought patterns (28). Exercise training relieves tension by stimulating the nervous system and regulating cortical excitability, and it can also induce changes in circulating endocannabinoids, promoting positive subjective emotions in patients (34). On the basis of emotional improvement, trimodal prehabilitation further alleviates fatigue symptoms through molecular mechanisms. Exercise inhibits the release of pro-inflammatory cytokines, induces anti-inflammatory effects via myokines, and optimizes mitochondrial energy metabolism, thereby systematically reducing the inflammatory burden and effectively improving CRF (35). Aerobic exercise effectively stimulates the hypothalamic-pituitary-adrenal (HPA) axis, promotes endorphin secretion, and regulates cortisol and adrenocorticotropic hormone (ACTH) levels, all of which help reduce the sensation of fatigue (36). Resistance exercise enhances neuromuscular metabolic capacity, increases muscle strength and endurance, positively affects microcirculation, accelerates carbohydrate metabolism, and promotes blood circulation, nutrient uptake, and metabolic waste removal, thereby improving fatigue levels in patients (37). Psychological intervention not only reduces CRF by regulating negative emotions but also improves fatigue by lowering inflammatory biomarkers, and patients with somatic diseases may derive greater anti-inflammatory benefits from psychological intervention (38). Furthermore, nutritional supplementation provides the material basis for improving physical function and relieving discomfort symptoms. This multi-level, systemic physiological regulation ultimately manifests as increased energy levels and reduced perception of fatigue in patients.

Exercise therapies can significantly enhance physical function and reduce feelings of fatigue in breast cancer patients (39). A yoga-centered rehabilitation paradigm enhances quality of life and markedly diminishes anxiety and depression symptoms (40). The distinctive benefit of yoga is that it is not solely an aerobic workout but a holistic intervention system that incorporates meditation, breath control, and relaxation techniques. In this study, 60% of patients actively chose yoga as their primary exercise method. This high selection rate reflects women’s preference for integrated mind-body intervention approaches. Various pre-rehabilitation programs, in addition to yoga, also exhibited beneficial effects. As an illustration, Oh’s team developed an innovative medical qigong program for patients with metastatic breast cancer. This program consisted of a 10-week exercise regimen that included gentle stretching, body movements in standing postures, meditation, and breathing exercises, all of which significantly enhanced the quality of life for this group (41). This method, incorporating exercise, breathing, and meditation, effectively meets patients’ need for holistic mind-body balance. It is an essential adjunct to conventional medical systems in improving the quality of life for patients in advanced stages of sickness, necessitating greater investigation into its application value in future studies.

Notably, the analgesic effect of trimodal prehabilitation on pain relief was limited, which may be attributed to the characteristics of pain associated with metastatic breast cancer. The possible reasons are as follows. First, cancer pain is mainly related to organic lesions such as bone metastases and tumor compression, and non-pharmacological interventions alone are unlikely to reverse this pathological process. Second, approximately one-third of the patients in this study had bone metastases. The mechanisms underlying bone metastasis-induced pain primarily involve bone destruction and cytokine-mediated hyperalgesia, which may require more targeted multimodal analgesic regimens (42). Third, the intervention period was only four weeks, which may have been insufficient to observe meaningful improvement in chronic cancer pain. These findings suggest that the response to trimodal prehabilitation varies across different cancer-related symptoms. Pain, as a symptom predominantly driven by organic pathology, may require more specialized analgesic approaches. In addition, the use of analgesics was not reported in this trial, which may affect the interpretation of the pain outcomes. Future studies should prospectively collect data on analgesic use, consider integrating pain management into the intervention protocol, or conduct stratified analyzes of intervention effects based on different pain types.

Previous studies have shown that the risk of death in breast cancer patients is significantly associated with quality-of-life domains, including physical, emotional, and role function (43). In this study, we preliminarily observed a longer median progression-free survival (PFS) in the observation group after trimodal prehabilitation intervention. However, PFS is a key prognostic indicator in advanced cancer, and its influencing factors are complex and diverse, involving tumor biology, treatment selection, genetic subtype, subsequent therapy, comorbidity management, and patient adherence, among other confounders. Because this study is a single-center, small-sample randomized controlled trial, we have not yet performed multivariate analysis for the difference in PFS between the two groups. Specifically, we did not adjust for or include in multivariate regression analysis potential confounders such as age, KPS score, number of metastatic lesions, sites of metastasis, treatment regimen, BRCA mutation status, and PD-L1 expression level. Therefore, we cannot fully rule out the independent interference of these factors on survival outcomes, nor can we definitively conclude that trimodal prehabilitation is an independent factor for prolonging PFS. Thus, the observed PFS benefit in this study should be considered preliminary and exploratory. High-quality studies with multicenter design, larger sample sizes, and long-term follow-up, along with multivariate Cox regression analysis to adjust for confounders, are still needed.

This study moves beyond the existing limitation of prehabilitation research, which has mostly focused on the preoperative period in early-stage breast cancer, by applying trimodal prehabilitation to patients with advanced triple-negative breast cancer receiving chemotherapy. Thus, it adds new evidence supporting trimodal prehabilitation as an adjunctive treatment for symptom management in this population. In terms of the intervention model, we innovatively developed a 4-week feasible program combining in-hospital guidance, home-based execution, and online supervision, while integrating mindfulness meditation and five-element music therapy, achieving both scientific rigor and cultural applicability. The results confirmed that trimodal prehabilitation alleviates physical and psychological symptoms in patients and provided preliminary insight into its effect on PFS. Regarding safety, continuous monitoring throughout the study showed that trimodal prehabilitation was well tolerated, with no serious adverse events or intervention-related withdrawals. The incidence of adverse events did not differ significantly between the observation and control groups, indicating that trimodal prehabilitation did not increase the risk of adverse events.

However, several limitations of this study must be acknowledged. First, the study only included patients with advanced triple-negative breast cancer, so the generalizability of the intervention effects to the broader breast cancer population across all molecular subtypes remains to be validated. Second, the intervention period was only four weeks, which prevented assessment of long-term effects or overall survival benefit. In addition, we did not perform multivariate regression analysis for PFS, and thus cannot exclude the influence of confounders on PFS or conclude definitively that trimodal prehabilitation is an independent factor for prolonged PFS. Third, this study only evaluated the observable effects of trimodal prehabilitation on patients and lacked objective data to explain the underlying mechanisms of its positive impact on advanced triple-negative breast cancer. Based on these limitations, future research should address the following areas. First, multicenter, large-scale, prospective randomized controlled trials should be conducted that include patients with advanced breast cancer of different molecular subtypes and that improve baseline assessments at enrollment to adjust for potential confounders and enhance the reliability of the conclusions. Second, the intervention protocol should be optimized by exploring different durations (e.g., 8 weeks or 12 weeks) and intensities of trimodal prehabilitation to identify the most effective model. Third, multi-omics technologies, including transcriptomics, metabolomics, and immunomics, should be integrated to gain deeper insight into the molecular mechanisms through which prehabilitation affects the inflammation-immune-tumor microenvironment. Fourth, follow-up should be extended to 1–3 years to systematically evaluate overall survival and the long-term maintenance of quality of life. Ultimately, a standardized and replicable intervention protocol should be established to provide evidence-based support for the comprehensive management of advanced breast cancer.

## Data Availability

The raw data supporting the conclusions of this article will be made available by the authors, without undue reservation.
